# A systematic review of artificial reefs as platforms for coral reef research and conservation

**DOI:** 10.1371/journal.pone.0261964

**Published:** 2022-01-21

**Authors:** Emily Higgins, Anna Metaxas, Robert E. Scheibling

**Affiliations:** 1 Department of Biology, Dalhousie University, Halifax, Nova Scotia, Canada; 2 Department of Oceanography, Dalhousie University, Halifax, Nova Scotia, Canada; University of Sydney, AUSTRALIA

## Abstract

Artificial reefs (ARs) have been used on coral reefs for ecological research, conservation, and socio-cultural purposes since the 1980s. We examined spatio-temporal patterns in AR deployment in tropical and subtropical coral reefs (up to 35° latitude) and evaluated their efficacy in meeting conservation objectives, using a systematic review of the scientific literature. Most deployments (136 studies) were in the North Atlantic and Central Indo-Pacific in 1980s – 2000s, with a pronounced shift to the Western Indo-Pacific in 2010s. Use of ARs in reef restoration or stressor mitigation increased markedly in response to accelerating coral decline over the last 2 decades. Studies that evaluated success in meeting conservation objectives (n = 51) commonly reported increasing fish abundance (55%), enhancing habitat quantity (31%) or coral cover (27%), and conserving target species (24%). Other objectives included stressor mitigation (22%), provision of coral nursery habitat (14%) or source populations (2%) and addressing socio-cultural and economic values (16%). Fish (55% of studies) and coral (53%) were the most commonly monitored taxa. Success in achieving conservation objectives was reported in 33 studies. Success rates were highest for provision of nursery habitat and increasing coral cover (each 71%). Increasing fish abundance or habitat quantity, mitigating environmental impacts, and attaining socio-cultural objectives were moderately successful (60–64%); conservation of target species was the least successful (42%). Failure in achieving objectives commonly was attributed to poor AR design or disruption by large-scale bleaching events. The scale of ARs generally was too small (m^2^ –10s m^2^) to address regional losses in coral cover, and study duration too short (< 5 years) to adequately assess ecologically relevant trends in coral cover and community composition. ARs are mostly likely to aid in reef conservation and restoration by providing nursery habitat for target species or recruitment substrate for corals and other organisms. Promoting local socio-cultural values also has potential for regional or global impact by increasing awareness of coral reef decline, if prioritized and properly monitored.

## Introduction

The global cover of scleractinian corals has declined dramatically since 1985 due to synergistic effects of increased ocean temperatures and acidification, predation, biological invasions, mechanical damage, and disease [1,2]. The increasing frequency and intensity of natural and anthropogenic stressors has altered coral reefs, contributing to large-scale phase shifts, in some regions, to alternative stable communities dominated by fleshy macroalgae [3,4], soft corals, corallimorpharia, or sponges [5]. It is estimated that more than 800 million people worldwide depend on coral reefs for food, coastal protection, and tourism [6–8], and that persistence of alternative stable states will cause a significant reduction in these ecosystem services [9].

Traditional conservation measures (e.g. no take-zones, reserves, and marine protected areas) have been used on coral reefs for decades [10–12], but attention has progressively shifted toward active restoration methods as a consequence of accelerating coral decline [13,14]. Ecological restoration is the process which assists the recovery of a degraded, damaged or destroyed ecosystem [15]. Since it may not be possible to remove the threat responsible for degradation or damage, the trajectory of recovery may allow adaptation to local and global changes [16]. The United Nations General Assembly recognized the pressing need to restore damaged ecosystems and proclaimed 2021–2030 to be the United Nations Decade on Ecosystem Restoration, with the primary goal being to prevent, halt and reverse the degradation of ecosystems worldwide. The United Nations Environment Assembly adopted a resolution that requested UNEP to specifically better define best practices for coral restoration [17]. Since the main threat to coral reefs is climate change [18], their restoration is likely most effective as a complementary tool in a larger management portfolio or as a temporary measure to minimize loss while global solutions are sought [17,19]. However, restoration of coral reefs has lagged behind and the spatial extent of restoration is the smallest compared to other major marine coastal ecosystems [20]. Thus, our knowledge on best practices for coral reef restoration is limited.

Motivations for coral reef restoration have ranged from ecological to cultural to legislative reasons, but experimental reasons appear to dominate [21,22]. Experimental approaches to active restoration include direct transplantation of corals, coral gardening, larval propagation, substrate manipulation, and substrate addition through the deployment of artificial reefs [17,19]. All approaches of active restoration have had certain shortcomings, such as short monitoring periods (average = 18 months) and small scales (< 100 m^2^) and have often lacked objectives [19]. Of these, artificial reefs (ARs), although popular for fish enhancement, have not been used as extensively for coral restoration [19], possibly because of the logistics of deployment and, on average, an order of magnitude greater cost than other approaches [20]. ARs have been deployed in coral ecosystems globally to address various conservation objectives, including enhancing fish and invertebrate biomass [23], increasing habitat quantity and structural complexity of denuded reefs [24,25], conservation of target species [26,27], and as nursery habitat for transplantation initiatives [28]. Examining the objectives of artificial coral reefs, success in meeting these objectives, and assessing their potential benefits as a restoration strategy can inform management decisions in different regions and under projected climate scenarios. However, for management decisions to be effective, the benefits of AR must be quantified and the efficacy of the methodologies (e.g. AR type, size, distribution, deployment location and period) evaluated.

ARs deployed in different temperate and tropical ecosystems can provide benefits to both benthic and pelagic communities [29] by supplying additional hard substrate for settlement [30], reducing fishing and tourism pressure on natural reefs [31], increasing heterogeneity of natural substrata [32,33], and providing shelter from predators and human disturbances [34,35]. As with other active restoration approaches, clearly defined objectives for the deployment of ARs are not always provided [29], presenting challenges with monitoring their effectiveness. There is also concern that the scale of ARs is too small to have long-term impacts on conservation or restoration of target species and their functional relationships [36]. It has been argued that ARs can introduce alien materials onto reefs that may harm the recipient community by leaking toxic compounds [37] or by scouring natural reefs if detached during coastal storms [38]. Additionally, there is debate as to whether ARs act as a source or sink for fish and invertebrate populations [39–42].

To assess the functional importance of ARs, an understanding of the dynamics of established benthic communities and their relationship with demersal and pelagic species is imperative [35]. Deploying ARs for restoration of coral ecosystems specifically is a relatively new strategy, and most research to date has been largely descriptive [43], with few replicated comparisons to natural reefs [44]. For example, there is increasing evidence that fish and invertebrate assemblages on ARs deployed in coral ecosystems do not mimic those on natural reefs [45–47]; the role of ARs in colonization by reef invertebrates is unknown [35]. Long-term data on species’ residence time, growth and survival, and production patterns on adjacent natural coral reefs rarely are collected during studies of ARs [34,40].

Planning AR deployments in coral ecosystems with specific goals and objectives coupled with long-term monitoring plans can allow the assessment of conservation outcomes from these interventions [17,19,29]. Here, we present results of a systematic review of the scientific literature focussed specifically on the use of ARs as an active restoration strategy for coral ecosystems. In particular, we examine stated objectives of ARs over the past 100 years and across 8 marine realms, along with records of the spatial scale, monitored taxa, and study duration. For studies that recorded progress toward meeting conservation objectives, we evaluate and discuss the reported success, and identify factors that may limit the attainment of objectives. Based on our findings, we propose that among all prospective conservation objectives for artificial coral reefs, the provision of nursery habitats and additional hard substrate for colonization, and the promotion of local socio-cultural values are those most likely to achieve conservation success. However, given the limited evidence of setting conservation objectives specific to deployment, the large variation in size, spacing and monitoring effort, and the potential cost, much more research is needed to assess the use of ARs as a coral restoration strategy globally.

## Methods

### Literature search and data extraction

We conducted searches in ISI Web of Science Core Collection (1900–2020), Scopus (https://www.scopus.com), and Google Scholar (https://scholar.google.ca) for peer-reviewed publications that measured or monitored ecological and socio-cultural variables on ARs deployed in tropical and subtropical coral reef ecosystems (up to 35° latitude). In each database, we adapted the following general search terms to account for syntax differences: (TITLE-ABS-KEY ((artificial* OR “man-made” OR construct*) W/2 (coral* OR reef* OR habitat* OR nursery*)) AND TITLE-ABS-KEY (coral* OR tropic* OR subtropic*)). The first two sets of search terms were optimized to return studies that incorporate AR structures that both were designed purposefully and became *de facto* ARs. The last set narrowed the scope of the search to articles pertaining to ARs deployed in coral ecosystems. Studies on both vertebrate and invertebrate groups were included. Searches in all databases were completed on 31 December 2020. To ensure scientific rigour in the assessment of conservation objectives, we did not include the 1000s of studies from the grey literature, the validity of which had not been evaluated through peer review.

**Fig 1:**
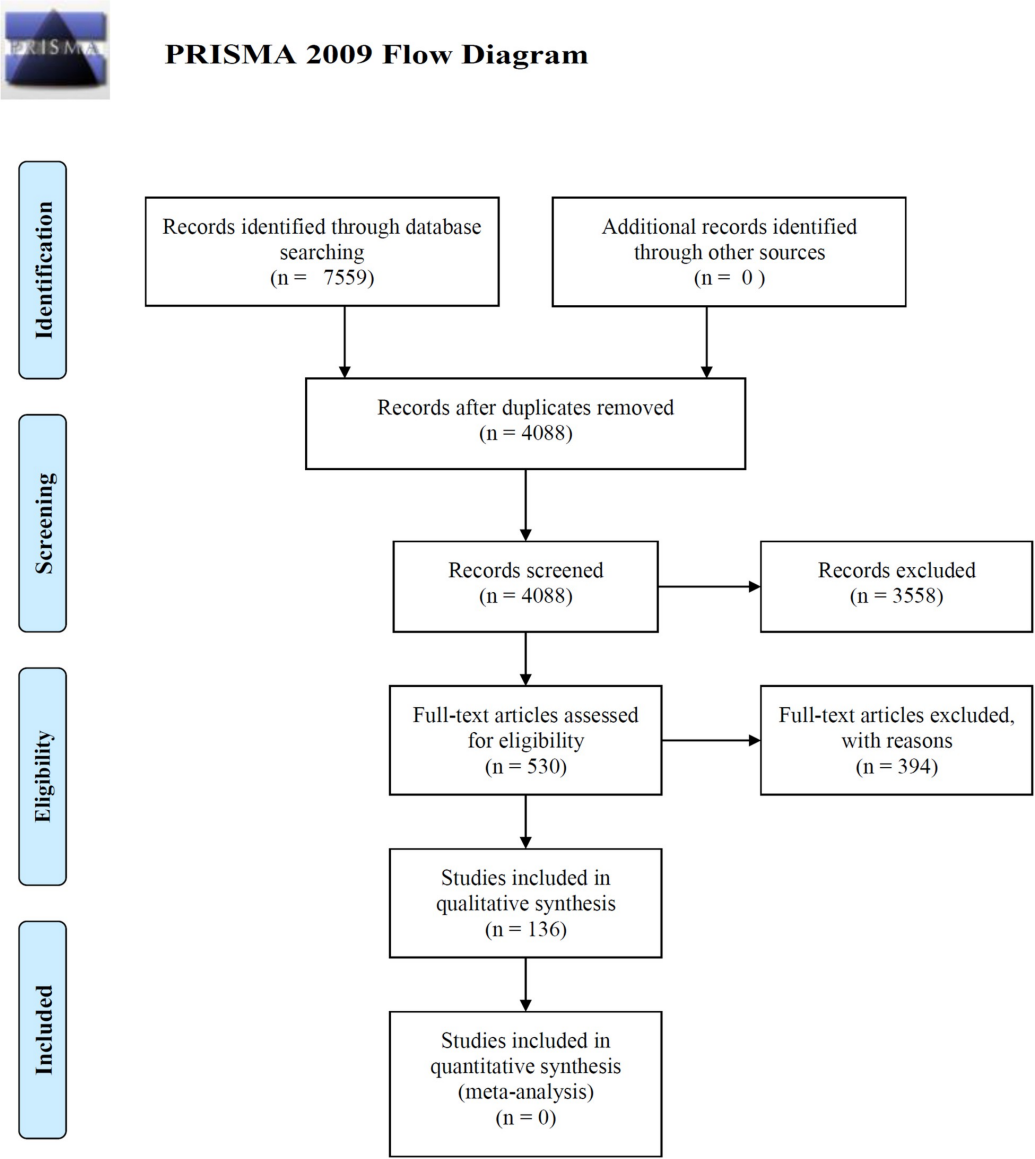
PRISMA 2009 flowchart describing the process of selecting articles for inclusion in the qualitative synthesis of our systematic review.

Over all databases, the search terms returned 4088 articles after duplicates were removed. All article citations and abstracts were imported into the web-based software review program Covidence (https://www.covidence.org), their titles and abstracts were screened, and 802 studies were extracted that included research on AR structures in coral reef ecosystems (Fig 1). A full text review was conducted for 530 articles, and data were extracted from 136 that met one or more of the following secondary inclusion criteria: 1) included a date, precise location, and depth of deployment, 2) included the precise dimensions and number of ARs in the study, and 3) stated an objective of AR deployment.

Articles were divided into two categories: 1) those that directly measured the success of meeting the objective(s) of ARs, and 2) those that were deployed for the purposes of scientific experiments or as *de facto* submergences (e.g. accidental ship groundings, dumping vehicles or building materials as waste). All 136 studies from both categories were surveyed for 1) duration of study, 2) clear description of AR dimensions, 3) targeted taxonomic groups, and 4) socio-cultural and ecological response variables used to assess whether the conservation objective(s) of the AR was being met. Latitude and longitude were extracted for each AR and then categorized into marine realms as defined in [48]. All 136 studies were used to examine spatio-temporal patterns of AR deployments as presented in the scientific literature. For the analyses of global AR abundance over time and to ensure representation of definitions used in the studies, we included all structures clearly defined as ARs by study authors and with a minimum area of ≥ 0.25 m^2^.

To ensure ecological relevance of conclusions about conservation success, studies reporting on progress towards attaining conservation objectives of deployed ARs fulfilled all secondary inclusion criteria listed above as well as two additional ones: (1) the monitored ARs were ≥ 1 m^2^ to allow for comparison with natural reef formations and knolls; and (2) for studies reporting on multiple ARs, individual AR structures were defined as such if they were at least 2 m from the nearest adjacent AR. This spacing reflects what is considered an AR by study authors and the methods used to ensure connectivity of motile organisms and larvae between ARs. It has been shown that ARs > 2 m apart can form distinct benthic communities [49]. A total of 53 studies fulfilled all inclusion criteria and were used in this analysis of conservation objectives.

### Classification of deployment objectives and response variables

Studies monitoring the success of an AR towards achieving one or more conservation objectives were further sub-classified into 8 categories of objectives: increase fish abundance, increase coral cover, conservation of target species (i.e. reef species of significant ecological or socio-cultural importance), socio-cultural value (e.g. economic evaluation, attractiveness to divers or tourists), serving as a source population for recruitment to the surrounding ecosystems, nursery or coral garden, increase habitat quantity, and stressor mitigation (i.e. deployment following catastrophic events, such as bleaching, severe tropical storms, and dredging). The ecological response variables used to assess success in meeting the conservation objective(s) of ARs were categorized according to the measurements (abundance, diversity, cover, recruitment, biomass, size distributions, survival/mortality, growth and reproduction rates, species turnover, connectivity/space use, and structural complexity) and by broad taxonomic groups (fish, coral, other invertebrates, and algae).

## AR deployments on coral reefs

### Definitions of AR

There is little standardization or agreement about the definition of AR in the scientific literature. Definitions within the studies examined in this review were disparate or absent. Authors reported on a vast array of structures, from *de facto* or accidental deployments to purposefully designed and deployed ARs. *De facto* or accidentally deployed ARs are wide ranging. Most are wrecks (or pieces of wrecks) of various numbers (15 in one case [50]), sizes and types of vessels; retired oil rigs [51], breakwaters and coastal jetties [52], and ropes in a tuna farm [53] were also considered ARs. Purposefully deployed ARs ranged from piles of rocks [54] or tires on the seafloor [55] to specifically engineered structures optimized for recruitment of target species for conservation, such as casitas or Autonomous Reef Monitoring Structures [28,56,57]. This is a similar range in structures and materials as for all ARs and is not particular to tropical reefs [58]. We used a broad AR definition when examining spatio-temporal patterns of AR deployment to accurately characterize the wide variety of structures that are currently being categorized as ARs in the peer-reviewed literature.

There is also little consistency in AR area within the peer-reviewed literature. Deployments of ARs for conservation purposes were conducted on a larger scale than ARs deployed for scientific experimentation. Most ARs used in experimental studies (70%) were 1–5 m^2^ (Table 1), while more than a half (60%) of ARs with conservation objectives were > 150 m^2^ (Table 2). The small size in experimental studies likely reflects logistical constraints of monitoring large reef structures in scientific experiments or of experimentally controlling and disentangling confounding abiotic effects of reef development on larger ARs [36]. Spacing between individual ARs is not well reported in studies examining structures with conservation objectives, which often neglect to distinguish between ARs and AR modules. Nearly all studies that monitored communities on *de facto* reefs reported that the structures were > 150 m^2^; only two studies monitored response variables on ARs of smaller area (Table 1).

**Table 1:** AR deployment for scientific experimentation or *de facto* ARs deposited as marine waste or accidental submergences (n = 75 studies).

Objective	No. of studies	Response variables	Study duration (y)	Reef area (m^2^)	Marine realm
Scientific experimentation	53	Fish density (23), fish diversity (13), fish recruitment (8), invertebrate density (8), coral recruitment (4), coral diversity (4), coral cover (4), coral size-distributions (1), invertebrate diversity (4), invertebrate recruitment (3), fish size-distributions (3), fish biomass (2), structural complexity (2), invertebrate biomass (2), coral density (2), coral survival/mortality (2), invertebrate survival/mortality (1), fish survival/mortality (2), invertebrate cover (2), coral growth (1), invertebrate growth (1), fish connectivity/ space use (2), species turnover (1), other ecological response variables (4)	1.8, 0.08–9	1–5 (37) 5–25 (6) 75–150 (4) >150 (6)	Tropical Atlantic (22) Western Indo-Pacific (12) Central Indo-Pacific (12) Eastern Indo-Pacific (2) Temperate N. Atlantic (2) Tropical E. Pacific (1) Temperate Australasia (2)
*De facto*	22	Fish diversity (10), fish density (10), coral cover (7), coral diversity (6), coral density (4), coral recruitment (2), coral size distributions (2), fish size distributions (3), fish connectivity/space use (1), invertebrate density (2), invertebrate diversity (1), invertebrate cover (1), fish biomass (1), coral survival/mortality (1), coral growth (1), coral genetics (1), socio-cultural variables (1), structural complexity (1), other ecological response variables (2)	1.9, 0.33–11	25–75 (1) 75–150 (1) >150 (22)	Western Indo-Pacific (9) Temperate N. Atlantic (5) Tropical Atlantic (5) Central Indo-Pacific (2) Temperate N. Pacific (1)

**Fig 2:**
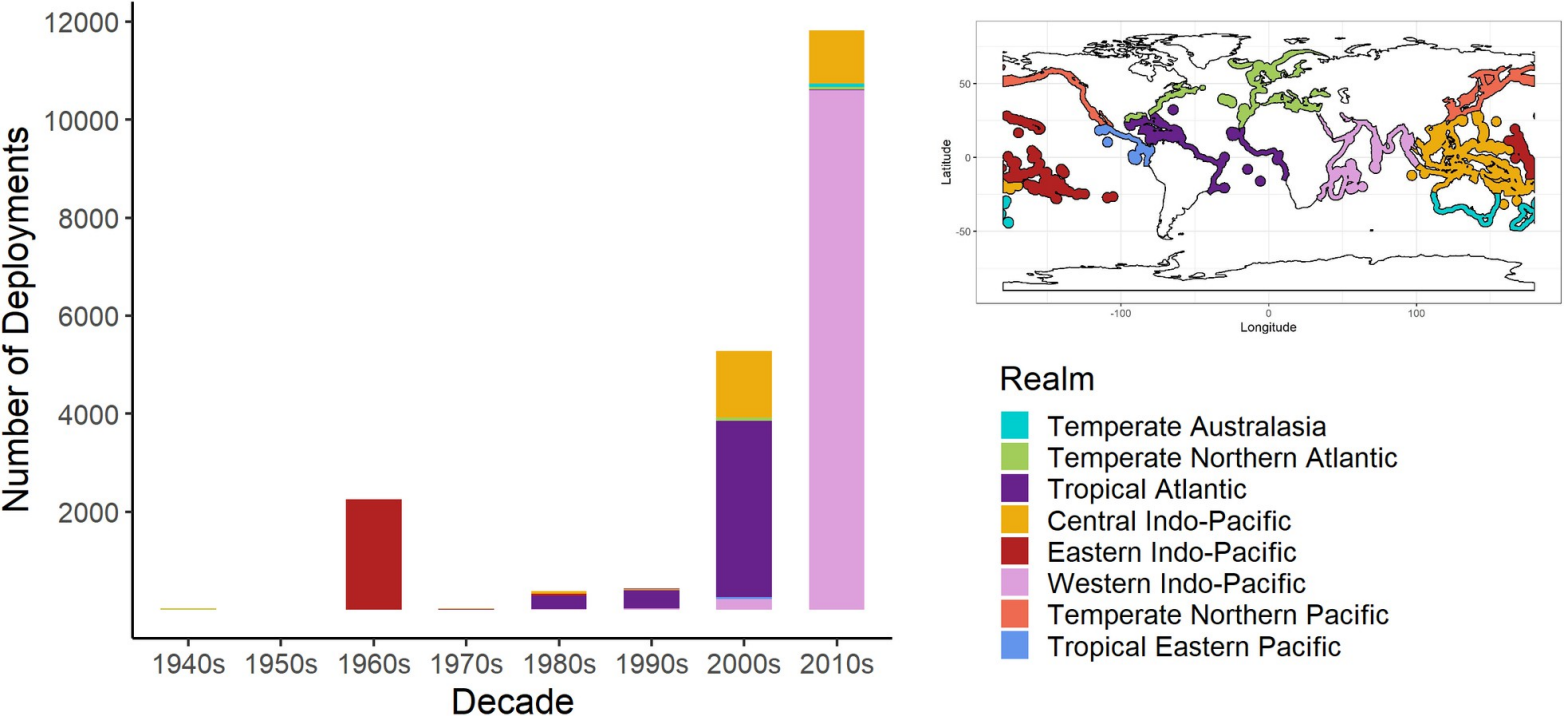
Abundance of tropical and subtropical (up to 35° latitude) deployments of ARs in each marine realm by decade of deployment from 1940s to 2010s. Inset shows marine realms defined by [48].

Response variables, study duration (mean, range), AR reef area, and marine realm (see Fig 2 for bioregions) are given for each objective. Numbers in parentheses are studies per variable or category. Note: Individual studies may have multiple response variables. See Appendix 1 in S1 File for reference list.

**Table 2:** AR deployment for conservation objectives (n = 51 studies).

**AR objective**	**No. of studies**	**Response variables that address objective**	**Study duration (y)**	**Reef area (m^2^)**	**Reported success rate (%)**	**Reported reasons for limited success**
Increase fish abundance	28	Fish density (21), diversity (20), biomass (4), size distribution (2), recruitment (1), connectivity/space use (3), socio-cultural variables (1), other ecological response variables (3)	1.4, 0.01–3.0	1–5 (1) 5–25 (4) 25–75 (6) 75–150 (7) >150 (15)	64	Poor design for target species (2), colonization interference by invasive species (1), extensive bleaching during study (1), conclusions made about another conservation objective (4), depth limitations (1), inconclusive data (1)
Increase coral cover	14	Coral diversity (5), recruitment (3), density (4), cover (2), growth (3), survival (5), biomass (1), reproduction (1), other ecological response variables (1)	2.5, 0.50–5.0	1–5 (1) 5–25 (3) 25–75 (5) 75–150 (4) >150 (2)	71	Extensive bleaching during study (1), conclusions made about another conservation objective (3)
Conservation of target species	12	Coral recruitment (6), fish recruitment (1), coral diversity (5), invertebrate diversity (2), fish diversity (3), fish density (4), coral density (6), invertebrate density (3), coral cover (2), invertebrate cover (3), coral size distribution (1), coral survival (2), coral growth (3), coral biomass (1), invertebrate biomass (1), fish biomass (1), coral reproduction (1), fish connectivity/space use (1)	2.0, 0.55–3.5	1–5 (1) 5–25 (2) 25–75 (4) 75–150 (4) >150 (1)	42	Poor design for target species (1), extensive bleaching during study (2), colonization interference by invasive species (1), interference by vessels (1)
**AR objective**	**No. of studies**	**Response variables that address objective**	**Study duration (y)**	**Reef area (m** ^**2**^ **)**	**Reported success rate (%)**	**Reasons for limited success**
Socio-cultural value	8	Diver behaviour and attitudes towards ARs (2), diving tourism/public education (4), cost-benefit analysis (1), citizen science (1), fishermen attitudes towards ARs (1)	2.5, 0.50–10	1–5 (2) 5–25 (1) 25–75 (2) >150 (3)	63	Conclusions made about another conservation objective (3)
Provide nursery area	7	Coral growth (4), reproduction (1), survival (5), fish density (1)	0.9, 0.08–2.0	1–5 (3) 5–25 (2) 75–150 (1) >150 (1)	71	Extensive bleaching during study (1), conclusions made about another conservation objective (1)
Increase habitat quantity	16	Coral recruitment (4), coral diversity (4), coral density (1), fish diversity (8), fish density (8), coral survival (5), coral growth (2), invertebrate biomass (1), invertebrate diversity (3), invertebrate cover (2), invertebrate density (3), invertebrate growth (1), fish biomass (2), fish connectivity/space use (3), fish recruitment (1), fish size distributions (1), structural complexity (1), socio-cultural variables (1), other ecological response variable (3)	1.8, 0.01–3.5	1–5 (1) 5–25 (2) 25–75 (1) 75–150 (6) >150 (4)	63	Extensive bleaching during study (2), depth limitations (1), conclusions made about another conservation objective (2), interference by vessels (1)
Stressor mitigation	11	Fish diversity (5), coral cover (4), fish density (4), coral recruitment (4), coral growth (2), coral survival (2), invertebrate density (3), invertebrate cover (1), invertebrate diversity (2), coral diversity (6), coral density (5), structural complexity (2), other ecological response variable (1)	2.6, 0.70–5.0	1–5 (4) 5–25 (1) 25–75 (2) 75–150 (1) >150 (3)	64	Extensive bleaching during study (2), conclusions made about another conservation objective (2)

Response variables, study duration (mean and range), AR reef area (5 levels), reported success rate (%), and reported reasons for limited success are given for each objective. Numbers in parentheses are studies per variable or category. Note: Individual studies may have multiple objectives and response variables. See Appendix 2 in S1 File for reference list.

### Spatio-temporal patterns in deployments of ARs

There were only 4 reports of AR deployments in the scientific literature until the mid-twentieth century. More than 2200 ARs were deployed in the 1960s, most in Hawaii (Fig 2). Comparatively few deployments were recorded from the 1970s to the 1990s, with a greater than 2-fold increase from the 1960s in the 2000s, followed by a similar increase between the 2000s and the 2010s (Fig 2). The increase in the 2000s corresponds to the increased focus on effects of climate change on coral reefs in the late 1990s following the first major global bleaching event in 1998 [59]. In the 2010s, > 10000 deployments were reported during a single study on the Indian shelf [60], resulting in the highest recorded number of ARs in coral ecosystems globally. These temporal patterns parallel those for ARs in other coastal ecosystems, reflecting a general global transition in AR research [61].

Following the large number of AR deployments in the Western Indo-Pacific, the Tropical Atlantic region has the next greatest number of AR deployments to date, with a coral restoration effort in Antigua contributing substantially to the region’s deployments (~ 3500 ARs deployed in 2004). However, the high abundance of ARs from the Tropical Atlantic is biased by a high intensity of study and frequency of publication from the southern United States (particularly Florida) from the 1960s onward [61,62]. Florida has a long history of AR deployment, with reefs often made from cheap waste materials (tires, metal construction materials, automotive parts) or *de facto* structures (sunken vessels, planes) [63].

The Central and Eastern Indo-Pacific exhibit similar numbers of AR deployments (Fig 2). AR deployments in the Eastern Indo-Pacific are attributed mostly to a single location (Hawaii), while in the Central Indo-Pacific they are distributed across several countries (e.g. Australia, Indonesia, Malaysia, Taiwan, Thailand, Vietnam) but largely concentrated in Indonesia. The regional interest of ARs in the Central Indo-Pacific may be a consequence of increasing exploitation of marine habitats [64] and the reliance of Southeast Asian countries on the economic value of ecosystem services associated with coral reefs (e.g. fisheries, tourism, shoreline protection) [65].

### Scientific experimentation and *de facto* AR deployments

Studies reporting on ARs that did not have a direct conservation-oriented objective were classified as either scientific experimentation or *de facto* submergences (Table 1) and were not included in our exploration of AR conservation objectives (Table 2). Over one third of the studies examined in this review (53 of 136) reported on scientific experiments conducted on ARs and 42% of these were conducted in the Tropical Atlantic realm. Overall, studies addressing only scientific objectives were marginally shorter than conservation-oriented projects, with mean durations of 1.7 y (Table 1) and 2.0 y (Table 2), respectively.

ARs recorded in peer-reviewed literature and deployed in the 1920s – 1950s were unplanned ship groundings that later were observed to have an AR effect by attracting fish and invertebrate colonizers [66]. Research efforts on *de facto* reefs (22 of 136 studies) reflect largely opportunistic monitoring, with data most often collected through digital imagery and a few manipulative experiments (Table 1). *De facto* ARs are the most variable in terms of study duration, ranging from 4 months to 11 years.

The Tropical Atlantic and Central Indo-Pacific realms have the highest number of AR deployments for scientific objectives or *de facto* deployments, with 414 and 402, respectively. In the past two decades, more than half (57%) of all such AR deployments are from the Central Indo-Pacific, a region which has experienced significant coral mortality since 1998 [4,67].

### Conservation-purposed AR deployments

#### Conservation objectives of Ars

**Fig 3:**
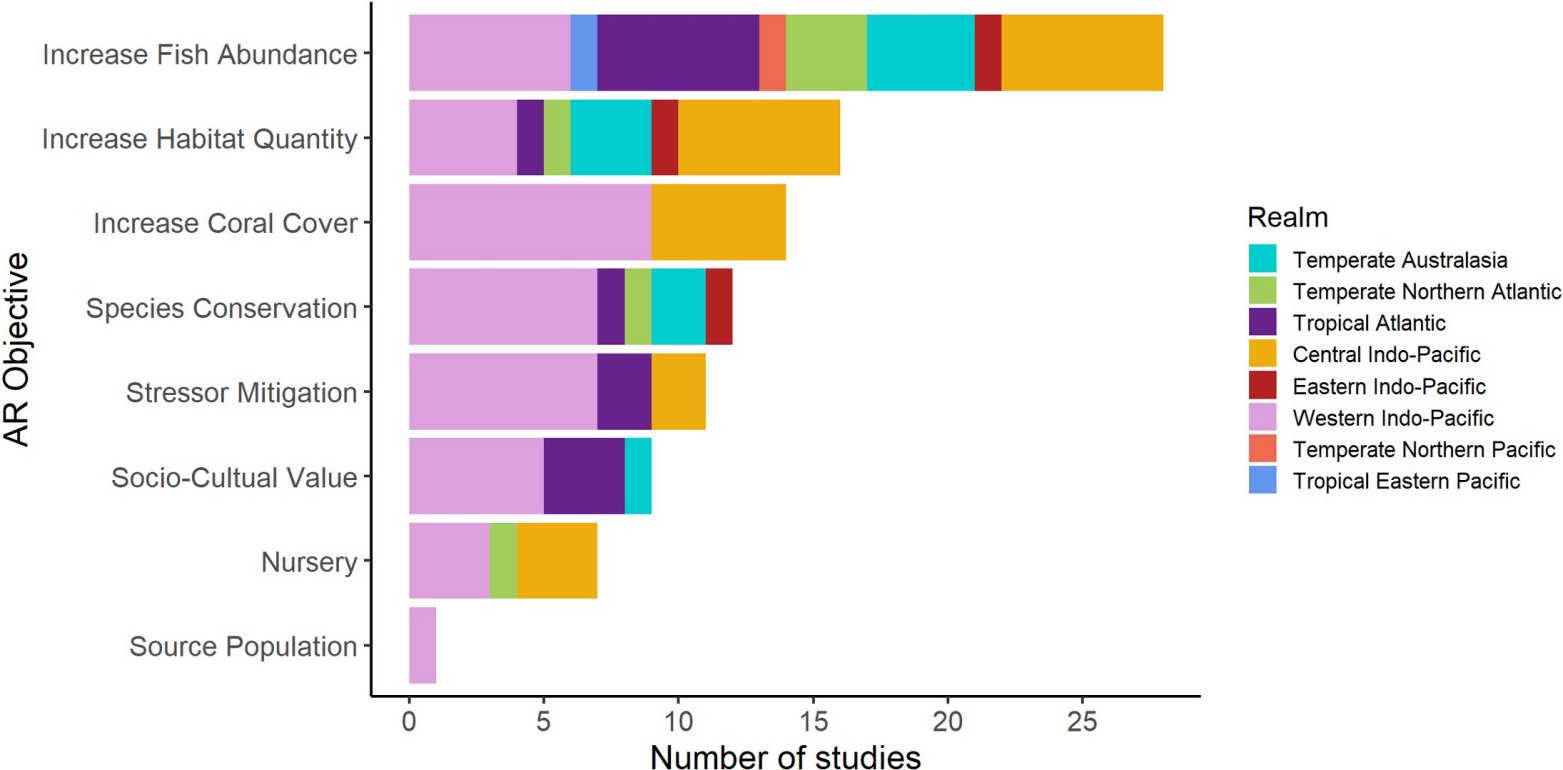
Number of studies that measured each objective by marine realm (Temperate Australasia, n = 11 studies; Temperate Northern Atlantic, n = 6; Tropical Atlantic, n = 12; Central Indo-Pacific, n = 22; Eastern Indo-Pacific, n = 3; Western Indo-Pacific, n = 42; Temperate Northern Pacific, n = 1; Tropical Eastern Pacific, n = 1) in ARs reporting success in meeting conservation objectives. See Fig 2 for map of bioregions.

The three most-commonly cited conservation objectives of ARs were increasing fish abundance (55%), increasing habitat quantity (31%), and increasing coral cover (27%) (Table 2). These conservation objectives were most common in the Western and Central Indo-Pacific, Tropical Atlantic and Temperate Australasia (Fig 3). Many of these ARs are in countries with substantial government funding for research and conservation, notably the USA (Florida) and Israel. In the Central Indo-Pacific, ARs with conservation objectives were predominantly deployed in countries with well-established national programs for AR development (Thailand, Malaysia), as well as those which received international funding in response to reef decimation by the Indian Ocean tsunami of 2004 (Thailand, Indonesia) [68,69].

The most frequently cited conservation objectives reflect a concentration on enhancing the quantity of hermatypic coral habitat and its most economically valuable inhabitants, including commercial fish (Fig 3). Fewer studies reported on ARs deployed for objectives related to the mitigation of natural and anthropogenic impacts on reef communities, such as conservation of target species (24%), mitigation of environmental stressors (22%), and provision of coral nurseries (14%), a relatively new restoration goal [70]. These AR conservation objectives are particularly common in the Central and Western Indo-Pacific (Fig 3). Studies addressing socio-cultural value and economic analyses on ARs (16%) were most frequently conducted in the Western Indo-Pacific. More specifically, 8 out of 51 studies were from the Middle East, where sea surface temperatures (SST) have increased more than 3 times the global average since 1985 [1]. This region is a global hotspot for AR research, leading the publication output in many categories of conservation objectives (Fig 3). Two studies (both from Malaysia) stated their conservation objective was to deter fishing trawlers and were not included in Table 2.

#### Taxonomic groups monitored on Ars

**Fig 4:**
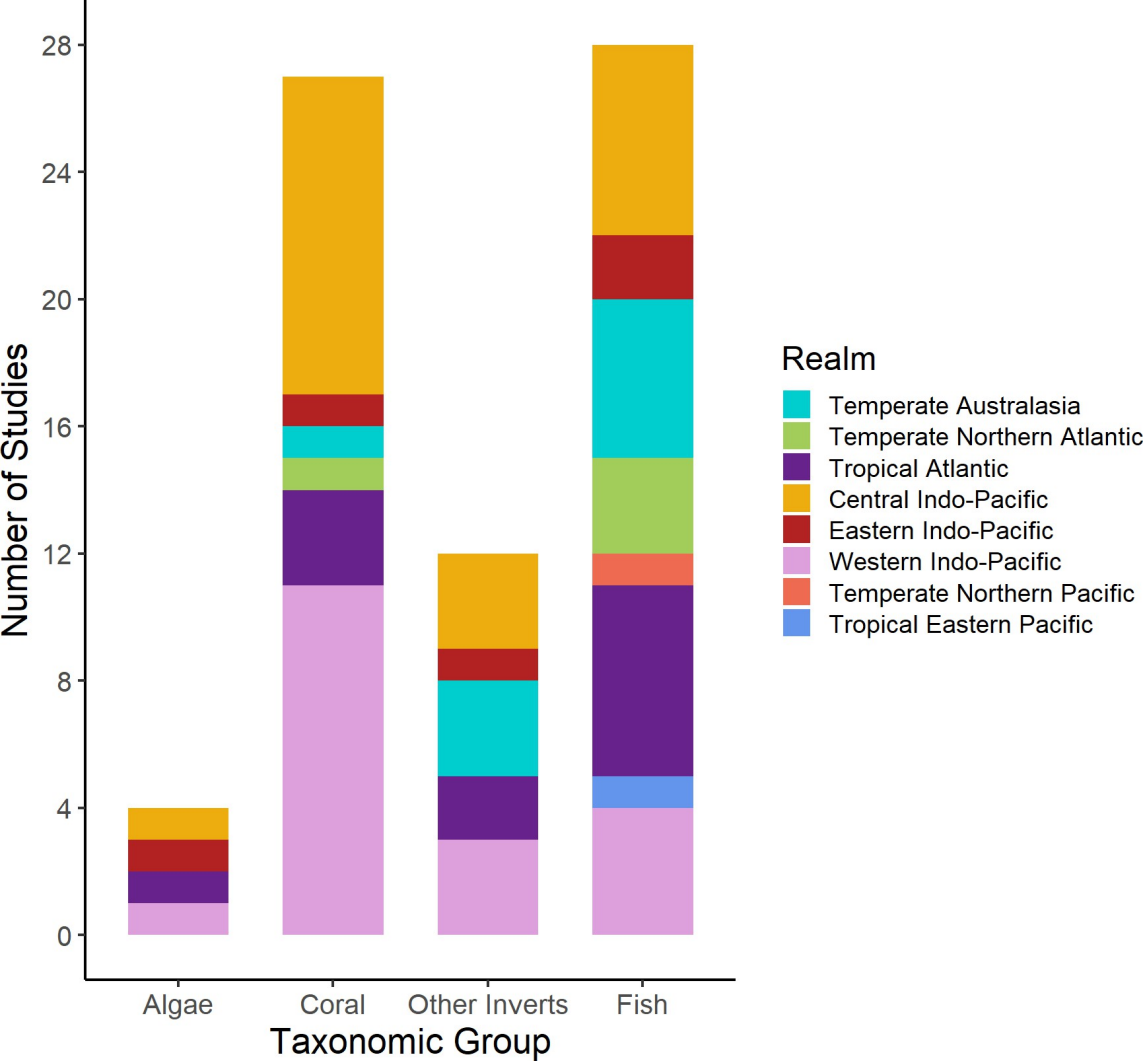
Number of studies monitoring conservation success of an AR (n = 51 studies) that measured ecological response variables for 4 taxonomic groups (Benthic Algae, Coral, Other Invertebrates, Fish) for each marine realm. See Fig 2 for map of bioregions.

Globally, fish and coral (29 and 26 studies, respectively) were the most frequently monitored taxonomic groups in the 51 studies assessing progress towards achieving conservation objectives of an AR. Most studies on corals (79%) were conducted in the Central and Western Indo-Pacific, while studies addressing fish populations were more evenly distributed across realms (Fig 4). Publications on corals were most frequent in the Central and Western Indo-Pacific, indicating biases in the AR conservation literature.

**Fig 5:**
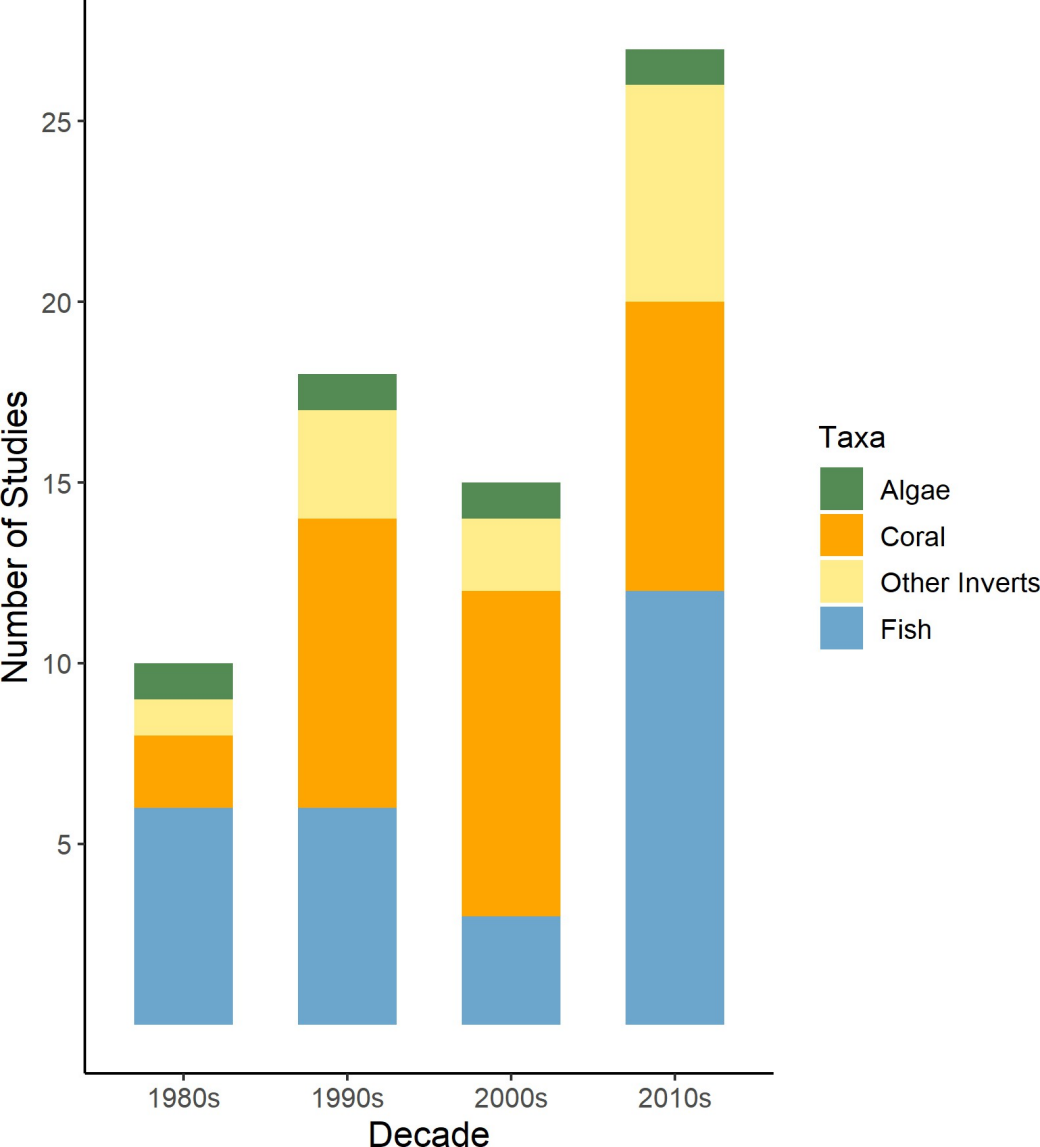
Number of studies monitoring conservation success of an AR (n = 51) that measured ecological response variables for 4 taxonomic groups (Benthic Algae, Coral, Other Invertebrates, Fish) on ARs by publication decade from 1980s to 2020s (to December 2020).

ARs have been deployed on coral reefs to assess and increase abundance of fish populations since the 1980s, and fish taxa were monitored in 55% of studies evaluating the conservation success of ARs (Fig 4). This is largely in response to declining fisheries on coral reefs due to overfishing and harmful fishing practices that have had catastrophic effects on coral reef fish since the 1980s, such as cyanide and dynamite fishing [4,71]. Many studies have focused on the population dynamics and behaviour of commercially or recreationally desirable fish species on and near ARs [72,73]. In the 1980s and 1990s, publications focused on protecting and increasing target fish species on reefs [74–76]. From the 1990s to 2010s, research effort on fish taxa has continually increased (Fig 5). In the late 2010s and 2020, a few conservation-oriented publications from Temperate Australasia and the Eastern Indo-Pacific focused on space use or connectivity of fish populations on ARs and adjacent natural reefs, likely reflecting an increased focus on the importance of connectivity in the persistence of reef fish assemblages [77–79].

Scleractinian corals were the other most frequently monitored (53%) taxonomic group on ARs in the peer reviewed literature. Similarly to fish population metrics, the number of studies monitoring coral communities increased every decade from the 1990s to 2010s (Fig 5), reflecting the increasing scale and severity of anthropogenic impacts on coral reefs [80,81]. Due to the alarming decline in coral cover and associated biodiversity worldwide, objectives of ARs that focus on coral conservation (e.g. coral nurseries or transplantation initiatives) [10,82] will likely continue to increase into the 2020s and beyond. More studies on coral conservation were published in the first five months of 2020 than during an entire decade in the 1980s and 1990s (Fig 5).

Benthic algae and invertebrates other than corals were the least monitored taxonomic groups on ARs (Fig 4). Understanding the successional patterns of these organisms on different AR structures is important because they can attract or deter target species [35]. Monitoring frequency of these underrepresented groups has increased since the 1990s, but they were still only measured in 0.05% (algae) and 20% (other invertebrates) of conservation studies published in the 2010s (Fig 5). However, despite increasing awareness of the importance of these groups for attaining conservation objectives of ARs, monitoring is still lacking in many regions [35,83]. Non-coral invertebrate groups were monitored in studies from the Indo-Pacific realms (16%), the Tropical Atlantic (17%), and Temperate Australasia (33%) (Fig 4). Only 1% of conservation studies measured benthic algae, all in the Indo-Pacific and the Tropical Atlantic. Fouling invertebrates and macroalgae growing on ARs can attract fish and motile invertebrate grazers [84–86]. Structures designed to support the growth of these organisms on coral reefs can enhance reef complexity and the abundance of local consumer populations [87,88]. Alternatively, excessive fouling by toxic invertebrates (e.g. ascidians and sponges) and some species of macroalgae deter coral larvae from settling and increase post-recruitment mortality rates [89–91]. Therefore, it is unclear whether ARs designed to promote fouling communities for the attraction of target fish species are conducive to coral recruitment.

## Potential of ARs as a conservation or restoration strategy on coral reefs

### Reported success of achieving AR conservation objectives

**Fig 6:**
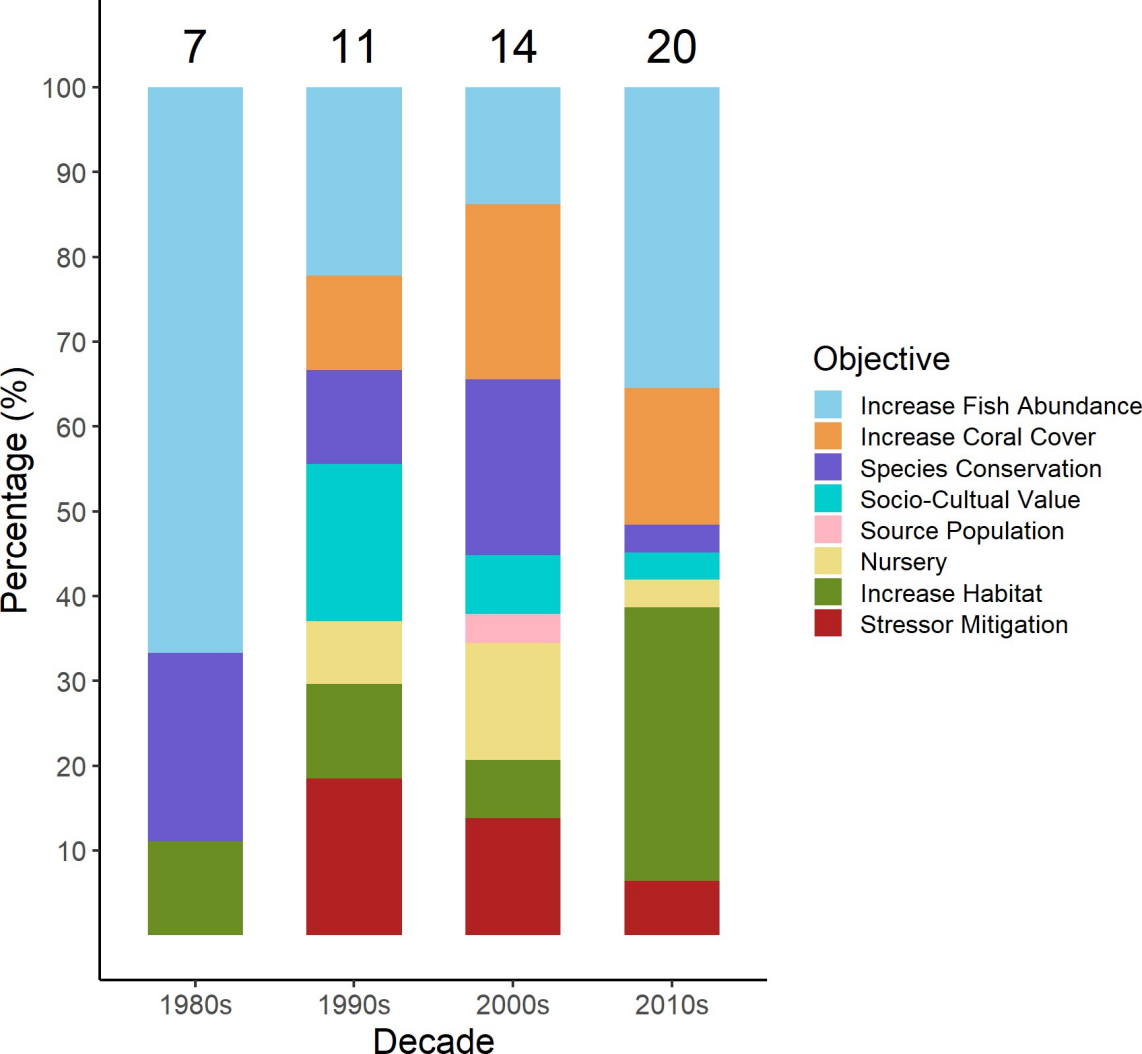
Percent of studies citing each category of AR conservation objective (n = 50 studies; 1 study was excluded because deployment date was unavailable) by deployment decade from 1960s to 2010s. Numbers above bars indicate number of studies.

Deployment of ARs with specific conservation objectives has varied over time (Fig 6) and geographic locations (Fig 3). Of the 51 studies, 65% reported success or progress towards achieving the conservation objective of AR deployment. Objectives with the highest reported rates of success were provision of nursery habitat and increasing coral cover (each 71%), followed by increasing fish abundance and mitigating effects of environmental impacts (each 64%), and increasing habitat quantity and attaining socio-cultural objectives (each 63%) and (Table 2). Conservation of target species was reported as successful in only 42% of studies. The most-commonly cited reasons for not achieving conservation objectives were poor AR design for target species and extensive bleaching during the study period (Table 2). Effective AR design considerations can be integrated into management strategies and deployment plans; however, reducing the level of extensive bleaching on artificial and natural reefs will require global cooperation for reducing carbon emissions [92].

Many studies reported multiple conservation objectives for each AR (Table 2), and 35% did not draw conclusions on all stated objectives. For example, if an AR was deployed for both increasing fish abundance and mitigating an environmental stressor, researchers may have recorded progress towards attaining only one of the two objectives due to constraints of logistics or expertise. Deploying ARs with multiple conservation objectives may reduce the likelihood of evaluating success or measuring ecological function of the AR. Structural design, site, and monitoring should be tailored for specific conservation objectives to limit ambiguous conclusions about success.

### Evaluation of reported success in achieving AR conservation objectives

**Fig 7:**
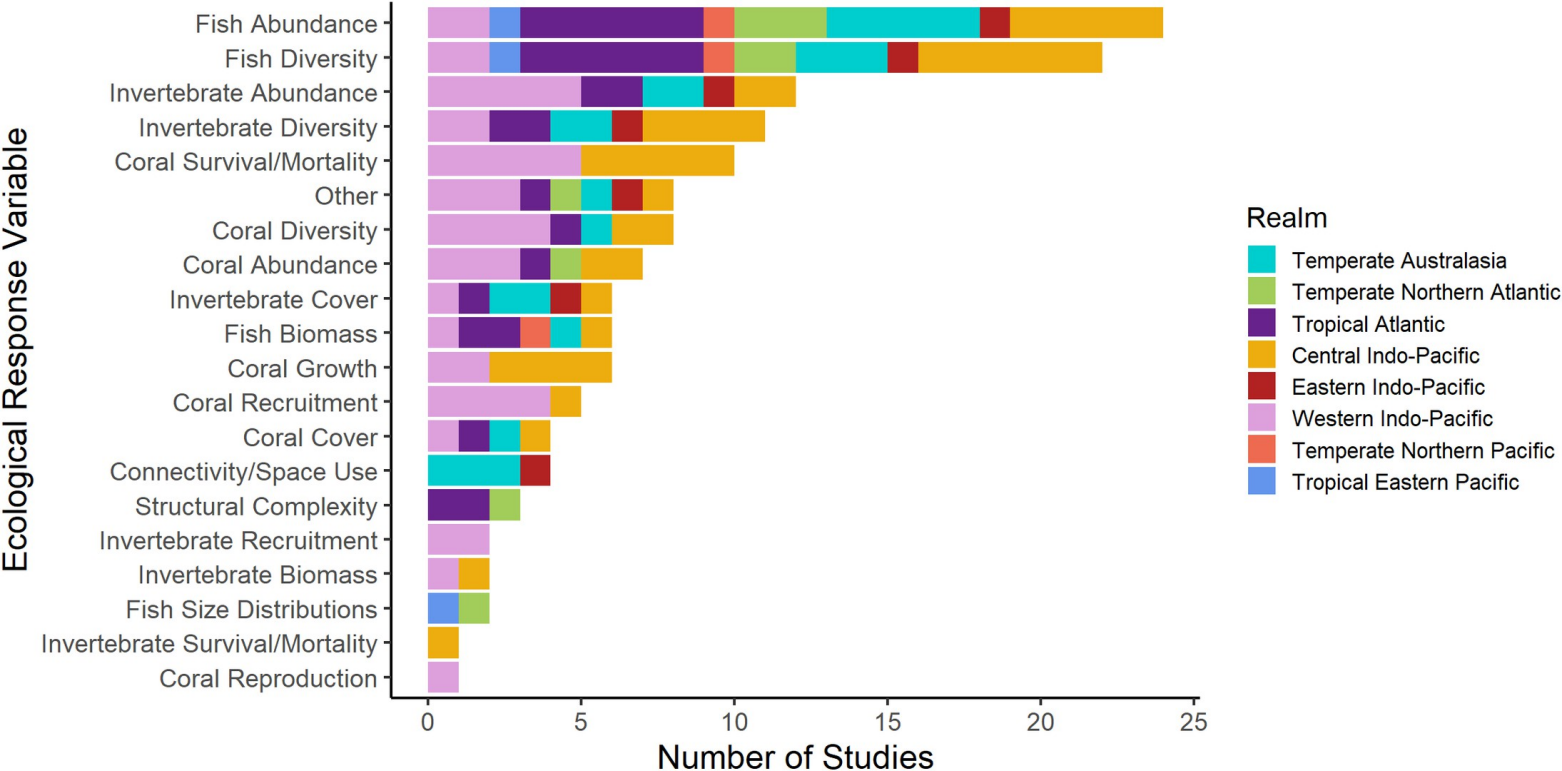
Number of studies that measured each ecological response variable by marine realm (Temperate Australasia, n = 6 study; Temperate Northern Atlantic, n = 4; Tropical Atlantic, n = 9; Central Indo-Pacific, n = 15; Eastern Indo-Pacific, n = 2; Western Indo-Pacific, n = 13; Temperate Northern Pacific, n = 1; Tropical Eastern Pacific, n = 1) in ARs reporting success in meeting conservation objectives. See Fig 2 for map of bioregions.

While ARs have been deployed to increase fish abundance since the 1980s, many studies monitoring their success did not measure appropriate ecological response variables for detecting increased fish production on the reef (Fig 7). For example, few studies examining the success of ARs in increasing fish abundance effectively monitored fish recruitment and movement between natural reefs and ARs. Therefore, authors were not able to distinguish whether ARs are attracting fish from adjacent habitats or enhancing abundance of resident populations. The three-dimensional structure and physical relief of the AR plays a significant role in attracting adult and juvenile fish from the water column [34,93,94]. Factors that contribute to the species composition of the colonizing fish community on ARs include distance from suitable substrate, distance from source populations, access by predators, access to food, and shelter for protection and egg-laying [34,63]. Disentangling whether ARs actually enhance production of fish or simply redistribute them within the ecosystem would enable researchers to evaluate whether ARs can be used to increase absolute fish abundance on coral reefs. This knowledge gap is well cited within the AR literature [34,40,42] and new approaches, such as modelling of biomass flux, may prove useful [95]. However, our results indicate that the gap remains poorly addressed in coral reef ecosystems specifically.

Increasing coral cover has been a relatively successful AR conservation strategy (Table 2). Overall, peer-reviewed studies used appropriate monitoring strategies for determining the success of this objective; however, there was regional variation in the measured response variables. Studies done in marine realms that encompassed ocean warming hotspots (Western and Central Indo-Pacific) concentrated on response variables pertaining to specific coral life history events (e.g. recruitment, survival/mortality, reproduction, and growth) (Fig 7). However, the scale of ARs has been too small to address regional losses in coral cover and the study duration has been too short to adequately assess a sustained increase in coral cover (Table 2), which can take decades to detect [96,97]. Small-scale rehabilitation projects using ARs to increase coral cover in denuded areas might be successful if proper design considerations and environmental stressors are taken into account [17]. For example, suspended ARs could be deployed on shallow water reefs and moved to deeper or cooler water during periods of peak SST to avoid bleaching [47].

Protecting select ecologically and socio-culturally important species was addressed through the objective of conserving target species. Authors reported limited success for this objective, with many studies citing inappropriate design for target species as the reason (Table 2). One study reported that colonization of target fish species was interrupted by the presence of invasive lionfish [98]. Structural design and site selection must be considered using species-specific requirements to increase the overall success of this conservation objective [17]. ARs deployed for the purpose of restoring, rehabilitating, or mitigating reef degradation for conservation of selected species need to be specifically engineered to enhance settlement and survival of targeted species [94].

Stressor mitigation has been increasingly used as a conservation objective for ARs over the past two decades (Fig 6). This is most likely a response to the increasing frequency and severity of coral bleaching events and concurrent climate change perturbations since the 1990s [1,80,99]. While this objective can be met on small spatial scales (e.g. preventing impacts of wave action and sedimentation) [60], our results suggest limited success when ARs are deployed to address ecosystem-wide stressors because ARs operate on a much smaller scale (m– 100s m) than natural reefs (10s – 100s km). Both scientific and conservation projects on ARs can be interrupted by large-scale bleaching events during the study period, making it difficult or impossible to assess the efficacy of ARs in mitigating stressors [26,68]. ARs do not directly alleviate underlying environmental stressors and may only be effective at remediating damages once the original perturbation has been substantially reduced or removed [17,100]. Coral reef restoration (including through the deployment of ARs) is most effective as an integrated component of wider management frameworks that include stressor mitigation [17]. The mismatch between the increasing spatial scale of stressors and the small scale of management interventions, such as ARs, reinforce the urgency for developing comprehensive management frameworks [101].

Arguably the most successful application of ARs is as nursery habitat for coral transplantation or source populations for which specific and appropriate ecological response variables (i.e. coral growth, reproduction, and survival) were used to determine success (Table 2). As long as coral colonies or fragments of colonies experience low mortality, increased larval production and a high yield of functional adult colonies with low environmental impact are possible [102,103]. Native species predicted to respond well to anticipated climatic changes can be selectively bred as a biological bank to re-populate natural reefs after disturbances [104]. If ARs are suspended or designed to detach from the seafloor, they also can be moved horizontally or vertically to avoid unfavorable growing conditions [70]. While nurseries operate on a relatively small scale compared to natural reefs, the likelihood of an AR functioning as a small source population in the region can be maximized by seeding it with high densities of coral species [28]. As with many studies published on active coral restoration strategies, publications examining the success of ARs as coral nurseries were exclusively from the Western and Central Indo-Pacific (Fig 3).

ARs deployed to increase habitat have been largely successful, likely because they add hard substrate to the benthic environment, making this a relatively attainable objective [39,105,106]. Measured response variables focused on benthic community development and fish presence at the AR (Table 2). Study durations for this objective were too short (0.01–3.5 y) to characterize success beyond initial recruitment and colonization phases for fish and invertebrates [63]. However, increasing hard substrate is not considered a high priority in reef conservation compared to addressing large-scale tissue loss of scleractinian corals caused by ocean acidification and warming [36].

In studies where deployment of ARs for socio-cultural purposes was the primary goal, the ARs were monitored appropriately and can be considered successful. However, in studies that combined socio-cultural and ecological objectives, conclusions were only drawn about the latter. Studies that monitored AR success using socio-cultural objectives employed a variety of socio-cultural variables, which can be separated into those monitoring human behaviour and emotions relative to ARs and those concerned with economic valuation (Table 2). In the Western Indo-Pacific, researchers surveyed the attractiveness of ARs to divers and diver behaviour on ARs [107]. Some studies examining the economic value of ARs lacked secondary inclusion criteria for this review but conducted a cost-benefit analysis [108] or estimated gross revenue generated from commercial fisheries as a consequence of ARs [109].

### Limitations of ARs and current knowledge gaps

Overall, our results indicate that ARs have limited success in meeting regional-scale conservation objectives, such as increasing abundance of coral and fish species or stressor mitigation. Nonetheless, these objectives are being increasingly cited in studies examining AR success, likely because of the acceleration of coral decline globally and the increasing call for remediating losses with active restoration strategies [14]. Because ARs mostly operate on a much smaller scale than natural reefs (except possibly small patch reefs), their success in addressing large-scale objectives must be assessed. Reference or control sites can provide context for the observed outcomes on ARs [29]. For example, a meta-analysis of 39 studies documented no difference in fish community metrics between natural and artificial reefs [41]. While it has been suggested that larger ARs (> 150 m^2^) support higher fish abundances [23], the extent to which ARs function as a source of fish production remains poorly understood [34,40,42]. Further, larger ARs are logistically difficult to fund, deploy, and monitor. The introduction of networks of ARs to regions with minimal environmental stressors may increase the success of abundance-oriented conservation objectives (i.e. increasing fish abundance and coral cover) by increasing colonizable reef area while fostering connectivity of fish and invertebrate species between degraded natural reefs [100]. Overall, small-scale objectives of ARs (e.g. increasing public education, selective coral breeding programs, training scientific and recreational divers) are far more achievable because they do not require additional intensive, long-term studies to determine their contribution to reef conservation and are generally successful when well defined and monitored.

Among all studies considered in this review, more than 73% spanned 3 years or less, which is too short a period for elucidating or predicting long-term shifts in coral reef populations. Studies that examined the success of ARs in meeting conservation objectives spanned 1 week to 5 years. This period matches the average for monitoring studies of several different coral restoration approaches [19,10] and may be adequate for addressing short-term goals of restoration at local scales [17]. For example, observation periods of months to years can allow monitoring colonization patterns in many short-lived organisms, such as reef-associated invertebrates (e.g. ascidians, bryozoans, and some sponges), that can settle, reproduce, and die on a substrate within months [47,110,111]. These durations also may be effective for monitoring fish populations on ARs, as many fish species have a life expectancy of under 5 years due to their inherent longevity or high rates of juvenile mortality [112–114]. Changes in coral community composition and dynamics, however, take much longer to detect [115,116]. For example, scleractinian coral communities require multidecadal monitoring to properly assess ecologically relevant trends in coral cover and species composition [96,97]. Longer monitoring periods also may be needed to capture effects of aperiodic or stochastic events, such as heatwaves or storms. Future studies examining the success of ARs in achieving coral-oriented conservation objectives must adjust study duration according to the relevant time scales of biotic and abiotic factors that govern the underlying ecological processes.

ARs can have some potentially negative impacts on the surrounding ecosystems. Often the materials used in ARs, such as rubber or plastics, are not biodegradable or may even leach toxic substances into the surrounding ecosystems [63]. Concrete, which is used for many ARs because of ease of production and low cost, in addition to leaching metals has a high alkalinity that may inhibit colonization [117,118]. To increase the ecological value of artificial structures, new materials using aggregate concrete with different chemistries are being developed [117,118]. ARs can also facilitate the introduction and spread of invasive species [119,120]; modification of the physical and chemical properties of the ARs and pre-seeding by native species may minimize colonization by non-native species [119]. Engineering solutions can provide potential mitigation strategies for the negative impacts of ARs.

## Conclusions and recommendations

1. To be an effective management tool, ARs deployed for conservation purposes must employ SMART (specific, measureable, achievable, realistic, time bound) objectives. Structural design, site, and monitoring should be tailored for specific conservation objectives to limit ambiguous conclusions about success. We showed that evaluation of success is less effective when ARs have multiple conservation objectives, either because some objectives are not evaluated or the measured ecological function is inappropriate for all objectives.
2. To be useful, ARs deployed for conservation purposes must be clearly described and accurately contextualized within the recipient environments to facilitate comparisons across geographic locations, target species and conservation objectives. Only 136 of 530 studies on research on coral reef ARs included a date, precise location, and depth of deployment, precise dimensions and number of ARs and stated an objective of AR deployment. In the scientific literature alone, the array of materials, shapes, sizes and distribution of ARs on the seafloor varied widely from haphazardly deployed sunken ships or car tires to deliberately deployed cinder blocks or reef balls.
3. The design of ARs must be suitable for the specific conservation objectives. Many of the studies we examined reported that ARs were unsuccessful in meeting their objective either because of an inappropriate design or because of loss of AR communities due to severe environmental perturbations, such as heatwaves. Future studies aiming to increase the efficacy of ARs for conservation purposes should choose structures and sites that are tailored for specific conservation objectives. The objective of using ARs to address ecosystem-wide restoration goals, such as increasing coral cover and stressor mitigation, has been met with limited success, because of the mismatch in scales between the AR and the recipient ecosystem. While nearly all AR projects are relatively small compared to adjacent natural reefs, they can address local, conservation-specific objectives, such as assisting the recovery of smaller patch reefs or local enhancement of coral cover if the stressor can be removed.
4. Monitoring of the performance of ARs in meeting conservation objective(s) must be based on ecologically relevant variables measured over appropriate time scales. For example, although increases in fish abundance and habitat quantity were the most frequently documented conservation objectives of ARs, monitoring over short-term scales failed to capture recruitment and community succession. Overall, fewer than one quarter of the 136 studies measured the success of ARs in meeting conservation objectives.

Based on their reported success as active restoration tools for tropical coral reefs, ARs are most likely to achieve their conservation objectives by providing nursery habitat for rearing target reef species or by supplying additional hard substrate for settlement and recruitment of corals and other marine organisms. We suggest that promoting local socio-cultural values also has potential for success if it is prioritized as an objective and properly monitored. This objective can be effective also globally, by increasing awareness of coral reef decline among tourists who mostly originate from countries without corals. While the effectiveness of ARs per se in achieving regional-scale conservation objectives may be limited, their integration into a larger restoration program could prove beneficial if used conjunction with other conservation strategies. However, given their relatively high cost, the implementation of ARs into larger restoration programs would require the development of better practices in identifying objectives, selecting the appropriate designs, and monitoring the relevant ecological responses.

## Supporting information

S1 Checklist PRISMA 2009 checklist. (DOC)

S1 File Literature used in Tables 1 and 2. (PDF)
