# Supplementary material for: A systematic review of artificial reefs as platforms for coral reef research and conservation
Source: PLoS One. 2022 Jan 21;17(1):e0261964. doi: 10.1371/journal.pone.0261964 (PMC8782470; doi:10.1371/journal.pone.0261964)
Supplement: S1 File — (PDF) [file pone.0261964.s002.pdf]

Appendix 1: Reference List for Table 1

Abelson A, Shlesinger Y. Comparison of the development of coral and fish communities on rock-aggregated artificial reefs in Eilat, Red Sea. ICES J Mar Sci. 2002; 59(Suppl):S122-6.

Akhwady R, Tamtomo PC, Luthfi OM. Used Evaluation of Stone Ash and Clamshell as Concrete Material of Artificial Reef at Pasir Putih Beach, Situbondo - Indonesia. Int J Appl Bas Sci. 2018; 18:8.

Albins MA, Hixon MA. Invasive Indo-Pacific lionfish *Pterois volitans* reduce recruitment of Atlantic coral-reef fishes. Mar Ecol Prog Ser. 2008; 367:233-8.

Alevizon WS, Gorham JC. Effects of artificial reef deployment on nearby resident fishes. B Mar Sci. 1989; 44(2):646-61.

Ammar MS, Mahmoud MA. A new innovated and cheap model in building artificial reefs. Egypt J Aquat Res. 2005; 31(1):105-117.

Ampou EE, Setiabudi GI, Widagti NR, Prasetia IND. Coral diversity on artificial reef from coconut shells in Northern Bali, Indonesia. Biodiversitas 2020; 21(9):4331-8.

Anker A. *Pseudalpheopsis guana* gen. nov., sp. nov. (Crustacea: Decapoda), a new alpheid shrimp from the British Virgin Islands, Caribbean Sea. Zool Stud. 2007; 46(4):428.

Atchison AD, Sammarco PW, Brazeau DA. Genetic connectivity in corals on the Flower Garden Banks and surrounding oil/gas platforms, Gulf of Mexico. J Exp Mar Biol Ecol. 2008; 365(1):1-2.

Baynes TW, Szmant AM. Effect of current on the sessile benthic community structure of an artificial reef. B Mar Sci. 1989; 44(2):545-66.

Belmaker J, Shashar N, Ziv Y. Effects of small-scale isolation and predation on fish diversity on experimental reefs. Mar Ecol Prog Ser. 2005; 289:273-83.

Belmaker J, Ziv Y, Shashar N. The influence of connectivity on richness and temporal variation of reef fishes. Landscape Ecol. 2011; 26(4):587-97.

Biesinger Z, Bolker BM, Marcinek D, Lindberg WJ. Gag (*Mycteroperca microlepis*) space-use correlations with landscape structure and environmental conditions. J Exp Mar Biol Ecol. 2013; 443:1-1.

Bohnsack JA. Species turnover and the order versus chaos controversy concerning reef fish community structure. Coral Reefs. 1983; 1(4):223-8.

Bohnsack JA, Harper DE, McClellan DB, Hulsbeck M. Effects of reef size on colonization and assemblage structure of fishes at artificial reefs off southeastern Florida, USA. B Mar Sci. 1994; 55(2-3):796-823.

Boland GS. Fish and epifaunal community observations at an artificial reef near a natural coral reef: Nineteen years at Platform High Island A389-A, from bare steel to coral habitat. In: McKay M, Nides J, Vigil D, editors. Proceedings: Gulf of Mexico fish and fisheries: Bringing together new and recent research; 2000 Oct; New Orleans, LA. New Orleans (LA): University of New Orleans; 2000. p. 372-392.

Brock RE, Kam AK. Focusing the recruitment of juvenile fishes on coral reefs. B Mar Sci. 1994; 55(2-3):623-30.

Bull AS, Kendall JJ. An indication of the process: offshore platforms as artificial reefs in the Gulf of Mexico. B Mar Sci. 1994; 55(2-3):1086-98.

Burt J, Bartholomew A, Bauman A, Saif A, Sale PF. Coral recruitment and early benthic community development on several materials used in the construction of artificial reefs and breakwaters. J Exp Mar Bio Ecol. 2009a; 373(1):72-8.

Burt J, Bartholomew A, Sale PF. Benthic development on large-scale engineered reefs: a comparison of communities among breakwaters of different age and natural reefs. Ecol Eng. 2011; 37(2):191-8.

Burt J, Bartholomew A, Usseglio P, Bauman A, Sale PF. Are artificial reefs surrogates of natural habitats for corals and fish in Dubai, United Arab Emirates? Coral Reefs. 2009b. 28(3):663-75.

Burt J, Feary D, Usseglio P, Bauman A, Sale PF. The influence of wave exposure on coral community development on man-made breakwater reefs, with a comparison to a natural reef. B Mar Sci. 2010; 86(4):839-59.

Burt JA, Feary DA, Cavalcante G, Bauman AG, Usseglio P. Urban breakwaters as reef fish habitat in the Persian Gulf. Mar Pollut Bull. 2013; 72(2):342-50.

Caley MJ, St John J. Refuge availability structures assemblages of tropical reef fishes. J Anim Ecol. 1996; 414-28.

Carassou L, Mellin C, Ponton D. Assessing the diversity and abundances of larvae and juveniles of coral reef fish: a synthesis of six sampling techniques. Biodivers Conserv. 2009; 18(2):355.

Cardoso AP, Matos MR, Rosa RS, Alvarado F, Medeiros AP, Santos BA. Increased fish diversity over day and night in structurally complex habitats of artificial reefs. J. Exp. Mar. Biol. Ecol. 2020; 522:151244.

Carr MH, Hixon MA. Artificial reefs: the importance of comparisons with natural reefs. Fisheries. 1997; 22(4):28-33.

Clarke RD. Effects of microhabitat and metabolic rate on food intake, growth and fecundity of two competing coral reef fishes. Coral Reefs. 1992; 11(4):199-205.

Colin PL, Laroche WA, Brothers EB. Ingress and settlement in the Nassau grouper, *Epinephelus striatus* (Pisces: Serranidae), with relationship to spawning occurrence. B Mar Sci. 1997; 60(3):656-67.

Connell SD. The relationship between large predatory fish and recruitment and mortality of juvenile coral reef-fish on artificial reefs. J Exp Mar Biol Ecol. 1997; 209(1-2):261-78.

Dos Santos DH, Silva-Cunha MD, Santiago MF, Passavante JZ. Characterization of phytoplankton biodiversity in tropical shipwrecks off the coast of Pernambuco, Brazil. Acta Bot Bras. 2010; 24(4):924-34.

Dupont JM, Hallock P, Jaap WC. Ecological impacts of the 2005 red tide on artificial reef epibenthic macroinvertebrate and fish communities in the eastern Gulf of Mexico. Mar Ecol Prog Ser. 2010; 415:189-200.

Edwards AJ, Guest JR, Heyward AJ, Villanueva RD, Baria MV, Bollozos IS, et al. Direct seeding of mass-cultured coral larvae is not an effective option for reef rehabilitation. Mar Ecol Prog Ser. 2015; 525:105-16.

Edwards RA, Smith SD. Subtidal assemblages associated with a geotextile reef in south-east Queensland, Australia. Mar Freshwater Res. 2005; 56(2):133-42.

Eggleston DB. Recruitment in Nassau grouper *Epinephelus striatus*: post-settlement abundance, microhabitat features, and ontogenetic habitat shifts. Mar Ecol Prog Ser. 1995; 124:9-22.

Eggleston DB, Grover JJ, Lipcius RN. Ontogenetic diet shifts in Nassau grouper: trophic linkages and predatory impact. B Mar Sci. 1998; 63(1):111-26.

Eggleston DB, Lipcius RN, Grover JJ. Predator and shelter-size effects on coral reef fish and spiny lobster prey. Mar Ecol Prog Ser. 1997; 149:43-59.

Eggleston DV, Lipcius RN, Miller DL. Artificial shelters and survival of juvenile Caribbean spiny lobster *Panulirus argus*: spatial, habitat, and lobster size effects. Fish B-NOAA. 1992; 90(4):691-702.

Eggleston DB, Lipcius RN. Shelter selection by spiny lobster under variable predation risk, social conditions, and shelter size. Ecology. 1992; 73(3):992-1011.

Enochs IC, Toth LT, Brandtneris VW, Afflerbach JC, Manzello DP. Environmental determinants of motile cryptofauna on an eastern Pacific coral reef. Mar Ecol Prog Ser. 2011; 438:105-18.

Epstein N, Bak RP, Rinkevich B. Strategies for gardening denuded coral reef areas: the applicability of using different types of coral material for reef restoration. Restor Ecol. 2001; 9(4):432-42.

Fennessy ST, Lotter P, Chater SC. Fish species composition and abundance on a subtropical, artificial reef on the east coast of South Africa. Afr Zool. 1998; 33(3):147-55.

Ferse SC. Artificial reef structures and coral transplantation: fish community responses and effects on coral recruitment in North Sulawesi/Indonesia [doctoral dissertation]. Bremen, Germany: University of Bremen; 2008.

Forrester GE, Steele MA. Variation in the presence and cause of density-dependent mortality in three species of reef fishes. Ecology. 2000; 81(9):2416-27.

Forrester GE. Strong density-dependent survival and recruitment regulate the abundance of a coral reef fish. Oecologia. 1995; 103(3):275-82.

Fowler AM, Booth DJ. How well do sunken vessels approximate fish assemblages on coral reefs? Conservation implications of vessel-reef deployments. Mar Biol. 2012; 159(12):2787-96.

Fox HE, Mous PJ, Pet JS, Muljadi AH, Caldwell RL. Experimental assessment of coral reef rehabilitation following blast fishing. Conserv Biol. 2005; 19(1):98-107.

Frederick JL. Post-settlement movement of coral reef fishes and bias in survival estimates. Mar Ecol Prog Ser. 1997; 150:65-74.

Giglio VJ, Luiz OJ, Schiavetti A. Recreational diver behavior and contacts with benthic organisms in the Abrolhos National Marine Park, Brazil. Environ Manage. 2016; 57(3):637-48.

Golani D, Diamant A. Fish colonization of an artificial reef in the Gulf of Elat, northern Red Sea. Environ Biol Fish. 1999; 54(3):275-82.

Gratwicke B, Speight MR. Effects of habitat complexity on Caribbean marine fish assemblages. Mar Ecol Prog Ser. 2005; 292:301-10.

Hata H, Hirabayashi I, Hamaoka H, Mukai Y, Omori K, Fukami H. Species-diverse coral communities on an artificial substrate at a tuna farm in Amami, Japan. Mar Environ Res. 2013; 85:45-53.

Hepburn LJ, Blanchon P, Murphy G, Cousins L, Perry CT. Community structure and palaeoecological implications of calcareous encrusters on artificial substrates across a Mexican Caribbean reef. Coral Reefs. 2015; 34(1):189-200.

Hernandez FJ, Shaw RF. Comparison of plankton net and light trap methodologies for sampling larval and juvenile fishes at offshore petroleum platforms and a coastal jetty off Louisiana. In American Fisheries Society Symposium 2003; Bethesda (MD): Am Fish S S; 2003. p.15-38.

Hixon MA, Beets JP. Shelter characteristics and Caribbean fish assemblages: experiments with artificial reefs. B Mar Sci. 1989; 44(2):666-80.

Hixon MA, Beets JP. Predation, prey refuges, and the structure of coral-reef fish assemblages. Ecol Monogr. 1993; 63(1):77-101.

Honório PP, Ramos RT, Feitoza BM. Composition and structure of reef fish communities in Paraíba State, north-eastern Brazil. J Fish Biol. 2010; 77(4):907-26.

Komyakova V, Swearer SE. Contrasting patterns in habitat selection and recruitment of temperate reef fishes among natural and artificial reefs. Mar Environ Res. 2019; 143:71–81.

Luthfi OM, Isdinato A, Sirait APR, Putranto TWC, Affandi M. Ecology of cubes artificial reef of Pantai Damas, East java, Indonesia. Eco Env Conserv. 2020; 26(4):1798-1805.

Marimuthu N, Ramachandran P, Sathish M, Dinesh N, Ramesh R. Recruitment pattern of Scleractinian coral spats on neighbouring artificial substrates at Kurusadai Reef Complex, Gulf of Mannar, India. Aquat Ecosyst Health Manag. 2019; 22:77–85.

Muzaki FK, Hanifa R, Akhwady R, Saptarini D, Buharianto. Short communication: Growth rate of Acropora muricata coral fragments transplanted on dome-shaped concrete artificial reef with different composition. Biodiversitas. 2019; 20:1555–9.

Paxton AB, Revels LW, Rosemond RC, Van Hoeck RV, Lemoine HR, Taylor JC, Peterson CH. Convergence of fish community structure between a newly deployed and an established artificial reef along a five-month trajectory. Ecological Engineering. 2018; 123:185–192.

Paxton AB, Christopher Taylor J, Peterson CH, Fegley SR, Rosman JH. Consistent spatial patterns in multiple trophic levels occur around artificial habitats. Mar Ecol Prog Ser. 2019; 611:189–202.

Pereira LC, Jiménez JA, Gomes PB, Medeiros C, da Costa RA. Effects of sedimentation on scleractinian and actinian species in artificial reefs at the Casa Caiada beach (Brazil). J Coastal Res. 2003; 418-25.

Perkol-Finkel S, Benayahu Y. Differential recruitment of benthic communities on neighboring artificial and natural reefs. J Exp Mar Bio Ecol. 2007; 340(1):25-39.

Perkol-Finkel S, Benayahu Y. The role of differential survival patterns in shaping coral communities on neighboring artificial and natural reefs. J Exp Mar Bio Ecol. 2009; 369(1):1-7.

Perkol-Finkel S, Zilman G, Sella I, Miloh T, Benayahu Y. Floating and fixed artificial habitats: effects of substratum motion on benthic communities in a coral reef environment. Mar Ecol Prog Ser. 2006; 317:9-20.

Perkol-Finkel S, Zilman G, Sella I, Miloh T, Benayahu Y. Floating and fixed artificial habitats: Spatial and temporal patterns of benthic communities in a coral reef environment. Estuar Coast Shelf S. 2008; 77(3):491-500.

Rilov G, Benayahu Y. Vertical artificial structures as an alternative habitat for coral reef fishes in disturbed environments. Mar Environ Res. 1998; 45(4-5):431-51.

Rilov G, Benayahu Y. Fish assemblage on natural versus vertical artificial reefs: the rehabilitation perspective. Mar Biol. 2000; 136(5):931-42.

Rilov G, Benayahu Y. Rehabilitation of coral reef-fish communities: the importance of artificial-reef relief to recruitment rates. B Mar Sci. 2002; 70(1):185-97.

Sreekanth GB, Lekshmi NM, Patil A. Performance of a shipwreck as an artificial fish habitat along Goa, west coast of India. Journ of Environ Biol. 2019; 40:170–176.

St. John J, Russ GR, Gladstone W. Accuracy and bias of visual estimates of numbers, size structure and biomass of a coral reef fish. Mar Ecol Prog Ser. 1990; 253-62.

Tupper M, Hunte W. Predictability of fish assemblages on artificial and natural reefs in Barbados. B Mar Sci. 1998; 62(3):919-35.

Wantiez L, Thollot P. Colonization of the F/V; Caledonie Toho 2 Wreck by a Reef-Fish Assemblage Near Noumea (New Caledonia). Atoll Res Bull. 2001; 485:1-19.

Wen CK, Chen KS, Hsieh HJ, Hsu CM, Chen CA. High coral cover and subsequent high fish richness on mature breakwaters in Taiwan. Mar Pollut Bull. 2013; 72(1):55-63.

Appendix 2: Reference List for Table 2

Adams AA. Fish assemblages associated with an established (more than 10 years old) artificial reef and an adjacent natural reef. In: Goodwin M, Acosta A. Proceedings of the 47th Annual Gulf and Caribbean Fisheries Institute; 1994 Nov; Nueva Esparta, Venezuela. Nueva Esparta (Venezuela): Proceedings of the 47th Annual Gulf and Caribbean Fisheries Institute. 2005; 613:441-457.

Al-Horani FA, Khalaf MA. Developing artificial reefs for the mitigation of man-made coral reef damages in the Gulf of Aqaba, Red Sea: coral recruitment after 3.5 years of deployment. Mar Biol Res. 2013; 9(8):749-57.

Ali A, Abdullah MP, Hazizi R, Marzuki AH, Hassan RB. Protecting Coastal Habitats and Enhancing Fisheries Resources Using Big Size Artificial Reefs in the East Coast of Peninsular Malaysia. Malays J Sci. 2013; 32:19-36.

Amar KO, Rinkevich B. A floating mid-water coral nursery as larval dispersion hub: testing an idea. Mar Biol. 2007; 151(2):713-8.

Arena PT, Jordan LK, Spieler RE. Fish assemblages on sunken vessels and natural reefs in southeast Florida, USA. In: Relini G, Ryland J. Biodiversity in Enclosed Seas and Artificial Marine Habitats. Proceedings of the 39th European Marine Biology Symposium; 21–24 Jul 2004; Genoa, Italy. Dordrecht (Netherlands): Springer; 2007. p. 157-171.

Bailey-Brock JH. Fouling community development on an artificial reef in Hawaiian waters. B Mar Sci. 1989; 44(2):580-91.

Becker A, Smith JA, Taylor MD, McLeod J, Lowry MB. Distribution of pelagic and epi-benthic fish around a multi-module artificial reef-field: Close module spacing supports a connected assemblage. Fish Res. 2019; 209:75–85.

Belhassen Y, Rousseau M, Tynyakov J, Shashar N. Evaluating the attractiveness and effectiveness of artificial coral reefs as a recreational ecosystem service. J Environ Manage. 2017; 203:448-56.

Blakeway D, Byers M, Stoddart J, Rossendell J. Coral colonisation of an artificial reef in a turbid nearshore environment, Dampier Harbour, western Australia. PLoS One. 2013; 8(9):e75281. https://doi.org/10.1371/journal.pone.0075281

Bortone SA, Shipp RL, Davis WP, Nester RD. Artificial reef development along the Atlantic coast of Guatemala. Gulf Mexico Sci. 1988; 10(1):4.

Campos JA, Gamboa C. An artificial tire-reef in a tropical marine system: a management tool. B Mar Sci. 1989; 44(2):757-66.

Chua CY, Chou LM. The use of artificial reefs in enhancing fish communities in Singapore. In: Sasekumar A, Marshall N, Macintosh DJ, editors. Ecology and Conservation of Southeast Asian Marine and Freshwater Environments including Wetlands. Dordrecht (Netherlands): Springer; 1994. p. 177-187.

Clark S. Artificial reef structures as tools for marine habitat restoration in the Maldives. Aquat Conserv. 1999; (14):197-202.

Clark S. Impacts of bleaching on coral communities on artificial reef structures in Maldives. In: Souter D, Obura D, Lindén O, editors. Coral reef degradation in the Indian Ocean. Stockholm (Sweden): CORDIO; 2000. p. 187-193.

Dahl KA, Patterson III WF, Snyder RA. Experimental assessment of lionfish removals to mitigate reef fish community shifts on northern Gulf of Mexico artificial reefs. Mar Ecol Prog Ser. 2016; 558:207-21.

Dupont JM. Artificial reefs as restoration tools: A case study on the West Florida Shelf. Coast Manage. 2008; 36(5):495-507.

Edwards AJ, Clark S, Zahir H, Rajasuriya A, Naseer A, Rubens J. Coral bleaching and mortality on artificial and natural reefs in Maldives in 1998, sea surface temperature anomalies and initial recovery. Mar Pollut Bull. 2001; 42(1):7-15.

Einbinder S, Perelberg A, Ben-Shaprut O, Foucart MH, Shashar N. Effects of artificial reefs on fish grazing in their vicinity: Evidence from algae presentation experiments. Mar Environ Res.2006; 61(1):110-9.

Florisson JH, Tweedley JR, Walker THE, Chaplin JA. Reef vision: A citizen science program for monitoring the fish faunas of artificial reefs. Fish Res. 2018; 206:296–308.

Froehlich CY, Kline RJ. Using fish population metrics to compare the effects of artificial reef density. PLoS One. 2015; 10(9):e0139444. https://doi.org/10.1371/journal.pone.0139444

Higgins E, Scheibling RE, Desilets KM, Metaxas A. Benthic community succession on artificial and natural coral reefs in the northern Gulf of Aqaba, Red Sea. PLoS ONE. 2019; 14(2): e0212842. https://doi.org/10.1371/journal.pone.0212842

Huntington BE, Lirman D. Species-area relationships in coral communities: evaluating mechanisms for a commonly observed pattern. Coral Reefs. 2012; 31(4):929-38.

Hylkema A, Debrot AO, Osinga R, Bron PS, Heesink DB, Isioka AK, Reid CB, Rippen JC et al. Fish assemblages of three common artificial reef designs during early colonization. Ecol Engineer. 2020; 157:105994

Irawan H, Yude R, Suhana MP, Suryanti A, Kurniawan D, Zahra A, et al. Assembled concrete reefs as a stand for coral transplantation on the seabed. IOP Conf Ser: Earth Environ Sci. 2019; 348:012046.

Isroni W, Samara SH, Santanumurti MB. Short Communication: Application of artificial reefs for fisheries enhancement in Probolinggo, Indonesia. Biodiversitas. 2019; 20(8):2273-78.

Jan RQ, Liu YH, Chen CY, Wang MC, Song GS, Lin HC, et al. Effects of pile size of artificial reefs on the standing stocks of fishes. Fish Res. 2003; 63(3):327-37.

Jayanthi M, Patterson Edward JK, Malleshappa H, Gladwin Gnana Asir N, Mathews G, Diraviya Raj K, Bilgi DS, Ashok Kumar TK, Sannasiraj SA. Perforated trapezoidal artificial reefs can augment the benefits of restoration of an island and its marine ecosystem. Rest Ecol. 2020; 28:233–243.

Jones ST, Asher JM, Boland RC, Kanenaka BK, Weng KC. Fish biodiversity patterns of a mesophotic-to-subphotic artificial reef complex and comparisons with natural substrates. PLoS ONE. 2020; 15(4):e0231668. https://doi.org/10.1371/journal.pone.0231668.

Khasanah RI, Herawati EY, Hariati AM, Mahmudi M, Sartimbul A, Wiadnya DGR, et al. Growth rate of acropora formosa coral fragments transplanted on different composition of faba kerbstone artificial reef. Biodiversitas. 2019; 20:3593–3598.

Komyakova V, Chamberlain D, Jones GP, Swearer SE. Assessing the performance of artificial reefs as substitute habitat for temperate reef fishes: Implications for reef design and placement. Sci Total Environ. 2019; 668:139–152.

Lalamentik LT, Kepel RC, Lumingas LJ, Rembet UN, Pratasik SB, Mantiri DM. Faviidae coral colonization living and growing on agricultural waste-materialized artificial substrate. AACL Bioflux. 2020; 13.

Lima JS, Zappes CA, Di Beneditto APM, Zalmon IR. Artisanal fisheries and artificial reefs on the southeast coast of Brazil: Contributions to research and management. Ocean Coast Manage. 2018; 163:372–382.

Munasik SA, Assyfa AN, Wijayanti DP, Irwani S, Pribadi R. Coral transplantation on a multilevel susbatrets of Artificial Patch Reefs: effect of fixing methods on the growth rate of two *Acropora* species. Biodiversitas 2020; 21(5):1816-22.

Perkol-Finkel S, Benayahu Y. Recruitment of benthic organisms onto a planned artificial reef: shifts in community structure one decade post-deployment. Mar Environ Res. 2005; 59(2):79-99.

Raj KD, Mathews G, Edward JKP. Long-term benefits of artificial reef modules for reef recovery in gulf of Mannar, Southeast India. J Coast Conserv. 2020; 24:53

Reeds KA, Smith JA, Suthers IM, Johnston EL. An ecological halo surrounding a large offshore artificial reef: Sediments, infauna, and fish foraging. Mar Environ Res. 2018; 141:30–38.

Romatzki SB. Influence of electrical fields on the performance of *Acropora* coral transplants on two different designs of structures. Mar Biol Res. 2014; 10(5):449-59.

Sammarco PW, Lirette A, Tung YF, Boland GS, Genazzio M, Sinclair J. Coral communities on artificial reefs in the Gulf of Mexico: standing vs. toppled oil platforms. ICES J Mar Sci. 2013; 71(2):417-26.

Sanguansil S, Yucharoen M, Samsuvan W. Diversity of fishes associated with artificial reefs off the coast of Sathing Pra, Songkhla. RIST. 2018; 1(1):16-21.

Seaman AN, Depper GL. Visiting scuttled ships: An examination of the important elements of the wreck diving experience. Tourism in Marine Environments. 2019; 14:31–44.

Selfati M, El Ouamari N, Lenfant P, Fontcuberta A, Lecaillon G, Mesfioui A, et al. Promoting restoration of fish communities using artificial habitats in coastal marinas. Biol Conserv. 2018; 219:89–95.

Setiadeswan R, Riyantini I, Mulyani Y, Pamungkas W. Community structure of macrozoobentos on artificial reef made from reef rubble and split rock in Tunda Island, province of Banten, Indonesia. World News of Natural Sciences. 2019; 27:128–140.

Shaish L, Levy G, Gomez E, Rinkevich B. Fixed and suspended coral nurseries in the Philippines: Establishing the first step in the “gardening concept” of reef restoration. J Exp Mar Biol Ecol. 2008; 358(1):86-97.

Shaish L, Levy G, Katzir G, Rinkevich B. Coral reef restoration (Bolinao, Philippines) in the face of frequent natural catastrophes. Restor Ecol. 2010; 18(3):285-99.

Simpson M, Coleman RA, Morris RL, Harasti D. Seahorse hotels: Use of artificial habitats to support populations of the endangered White’s seahorse Hippocampus whitei. Mar Environ Res. 2019; 157(104861).

Suyatna I, Saptura NA, Eryati R, Adnan A, Effendi M, Hanjoko T, et al. Fish colonization of the artificial reef at Gusung Batu Lampe Muara Badak, Kutai Kartanegara, East Kalimantan, Indonesia: A preliminary study [version 1; peer review: 1 not approved]. F1000Research. 2019; 8.

Thanner SE, McIntosh TL, Blair SM. Development of benthic and fish assemblages on artificial reef materials compared to adjacent natural reef assemblages in Miami-Dade County, Florida. B Mar Sci. 2006; 78(1):57-70.

Tran PD, Pham TV, Nguyen LT, Tran HV, Nguyen KQ. Artificial coral reefs restore coastal natural resources. Int J Fish Aquat. 2019; 7(3):128-33.

van Treeck P, Schuhmacher H. Initial survival of coral nubbins transplanted by a new coral transplantation technology-options for reef rehabilitation. Mar Ecol Prog Ser. 1997; 150:287-92.

van Treeck P, Schuhmacher H. Artificial reefs created by electrolysis and coral transplantation: an approach ensuring the compatibility of environmental protection and diving tourism. Estuar Coast Shelf S. 1999; 49:75-81.

Walker SJ, Schlacher TA. Limited habitat and conservation value of a young artificial reef. Biodivers Conserv. 2014; 23(2):433-47.
